# Supplementary figures and images for: Interleukin 6 is increased in preclinical HNSCC models of acquired cetuximab resistance, but is not required for maintenance of resistance
Source: PLoS One. 2020 Jan 8;15(1):e0227261. doi: 10.1371/journal.pone.0227261 (PMC6948745; doi:10.1371/journal.pone.0227261)

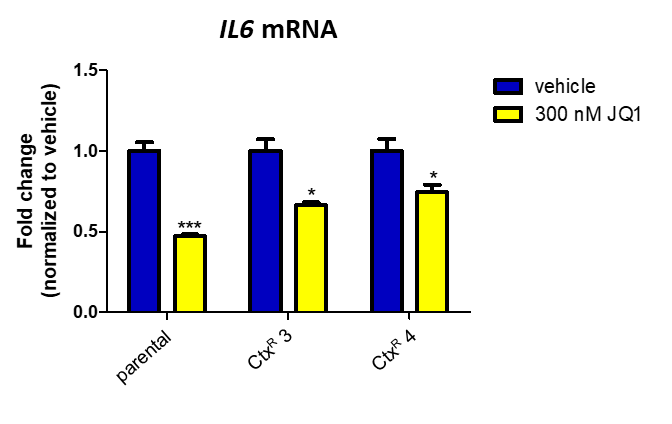

Supplement: S1 Fig — PE/CA-PJ49 parental, CtxR 3, and CtxR 4 cells were treated with vehicle (DMSO) or 300 nM JQ1. After 96 hours of treatment, RNA was extracted and qPCR was conducted using the IL6 primers listed in S1 Table (normalized to TBP). n = 3. *p<0.05; ***p<0.001. (TIF) [file pone.0227261.s001.tif]

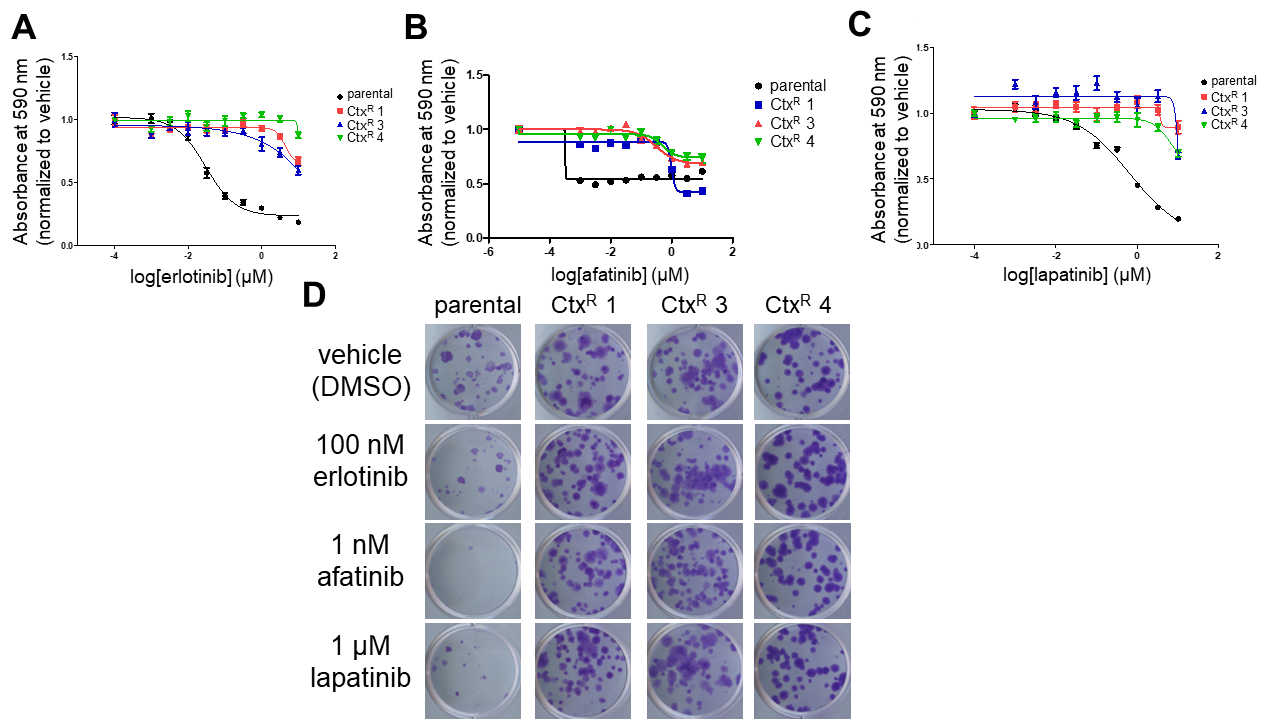

Supplement: S2 Fig — A, B, C) Erlotinib (A), afatinib (B), and lapatinib (C) dose response assays in PE/CA-PJ49 parental cells and CtxR clones treated for 96 h, then stained with crystal violet. n = 6. D) PE/CA-PJ49 parental cells and CtxR clones were plated at low density and treated with vehicle (DMSO), 100 nM erlotinib, 1 nM afatinib, or 1 μM lapatinib, then stained with crystal violet after 12 days of treatment. Media containing vehicle or drug was changed every four days. n = 4. (TIF) [file pone.0227261.s002.tif]

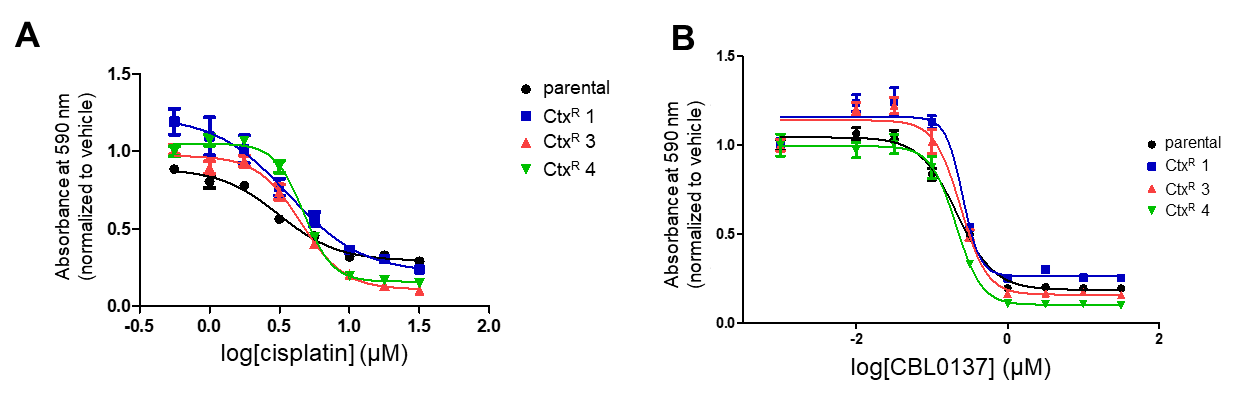

Supplement: S3 Fig — A, B) Cisplatin (A) and CBL0137 (B) dose response assays in PE/CA-PJ49 parental and CtxR cells treated for 96 h, then stained with crystal violet. n = 6. (TIF) [file pone.0227261.s003.tif]

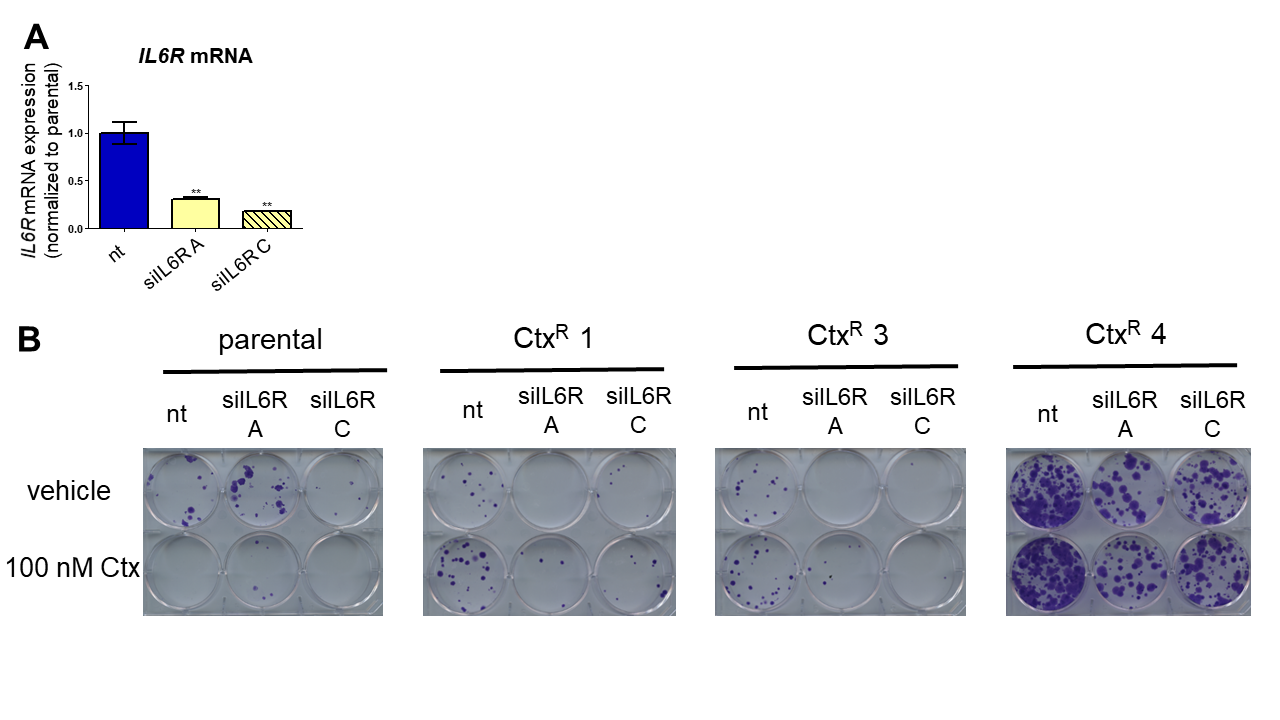

Supplement: S4 Fig — A) PE/CA-PJ49 parental cells were transfected with 10 nM nontargeting (nt) siRNA or one of two siRNAs targeting IL6R (siIL6R A and C). RNA was extracted 96 hours post-transfection and qPCR was conducted using the IL6R primers listed in S1 Table (normalized to TBP). n = 3. **p<0.01. B) PE/CA-PJ49 parental and CtxR cells were plated at a low density and transfected with 10 nM siRNA the next day. On the following day, and every four days thereafter, the cells were treated with vehicle (PBS) or 100 nM Ctx. The cells were stained with crystal violet 13 days post-transfection. (TIF) [file pone.0227261.s004.tif]

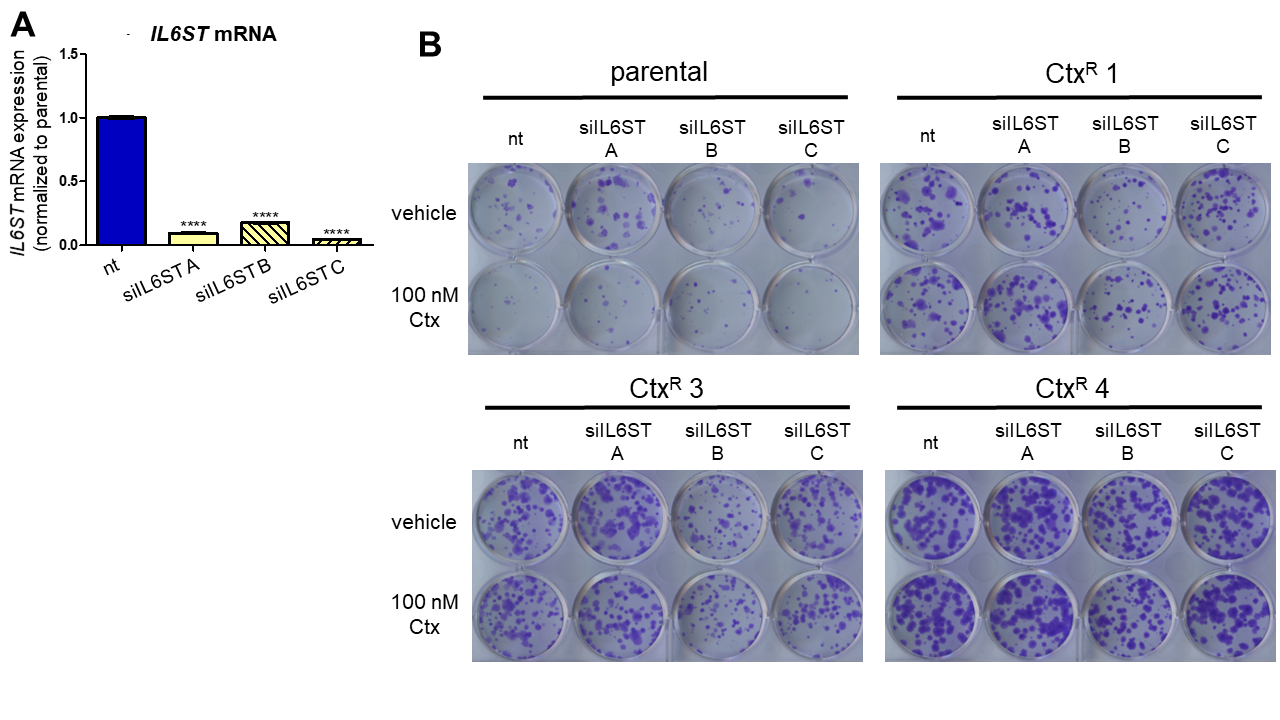

Supplement: S5 Fig — A) PE/CA-PJ49 parental cells were transfected with 10 nM nontargeting (nt) siRNA or one of three siRNAs targeting IL6ST (siIL6ST A, B, and C). RNA was extracted 96 hours post-transfection and qPCR was conducted using the IL6ST primers listed in S1 Table (normalized to TBP). n = 3. ****p<0.0001. B) PE/CA-PJ49 parental and CtxR cells were plated at a low density and transfected with 10 nM siRNA the next day. On the following day, and every four days thereafter, the cells were treated with vehicle (PBS) or 100 nM Ctx. The cells were stained with crystal violet 13 days post-transfection. (TIF) [file pone.0227261.s005.tif]

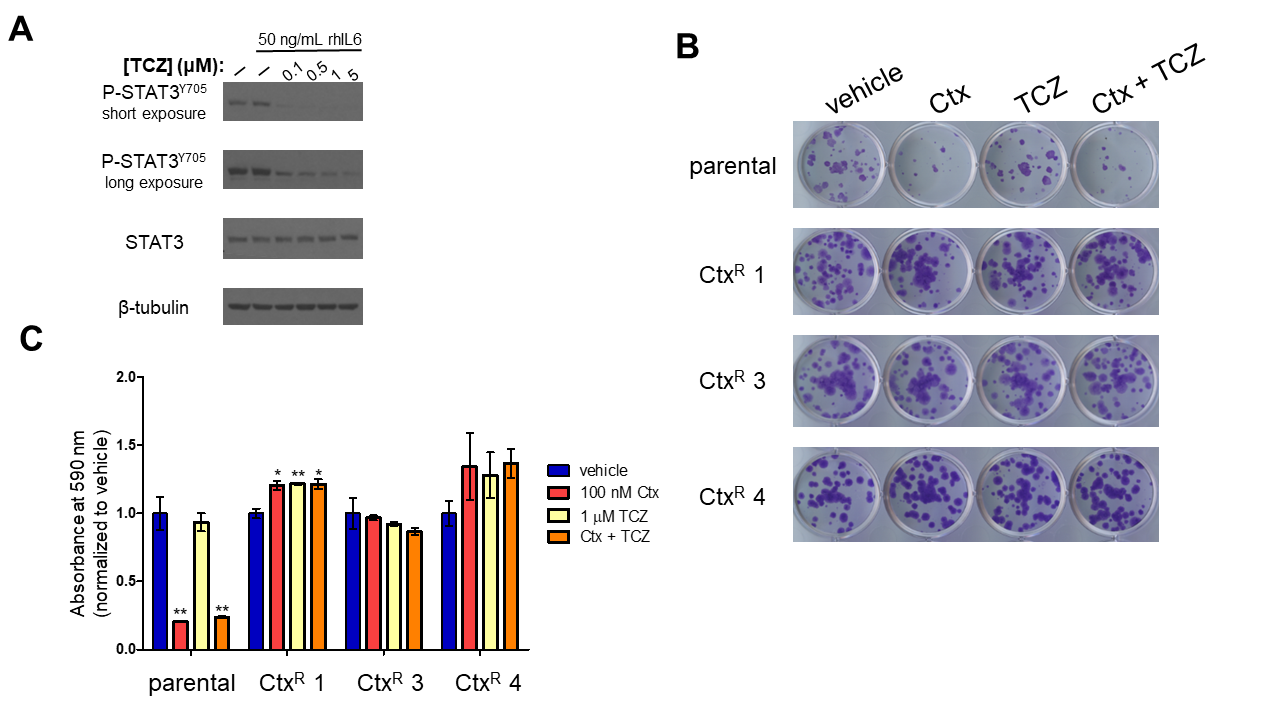

Supplement: S6 Fig — A) Serum-starved PE/CA-PJ49 parental cells were pre-treated for 2 hours with vehicle (PBS) or 100 nm– 5 μM TCZ, then treated with 50 ng/mL rhIL6 for 15 minutes. Cells were lysed in RIPA buffer and immunoblot was performed. β-tubulin image shown is from the STAT3 blot. B) PE/CA-PJ49 parental and CtxR cells were plated at a low density, then treated with vehicle (PBS), 100 nM Ctx, 1 μM TCZ, or the combination of Ctx and TCZ every 4 days. After a total of 12 days of treatment, the cells were stained with crystal violet. C) Crystal violet-stained cells from (B) were solubilized and absorbance at 590 nm was measured. Student’s two-tailed t-test was used to determine whether differences in absorbance at 590 nm were statistically significant (compared to vehicle-treated cells). n = 3. *p<0.05; **p<0.01. (TIF) [file pone.0227261.s006.tif]

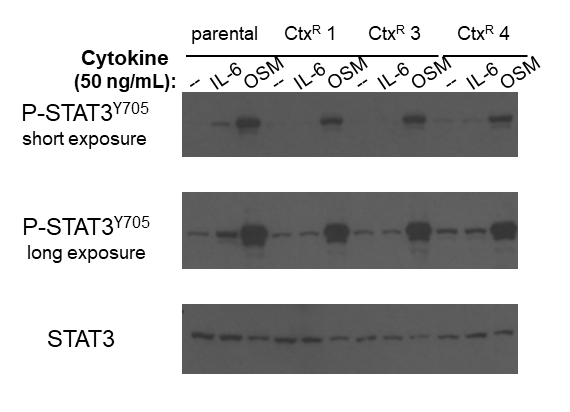

Supplement: S7 Fig — PE/CA-PJ49 parental and CtxR cells were serum starved for 4 hours, then treated for 15 minutes with 50 ng/mL rhIL6 or rhOSM. Cells were lysed in RIPA buffer and immunoblot was performed. (TIF) [file pone.0227261.s007.tif]

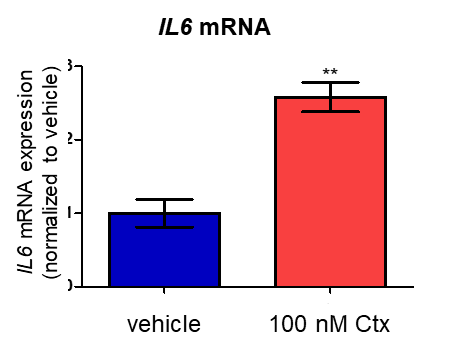

Supplement: S8 Fig — PE/CA-PJ49 parental cells were treated with vehicle (PBS) or 100 nM Ctx. After 96 hours of treatment, RNA was extracted and qPCR was conducted using the IL6 primers listed in S1 Table (normalized to TBP). Student’s two-tailed t-test was used to determine whether differences in IL6 expression were statistically significant. n = 3. **p<0.01. (TIF) [file pone.0227261.s008.tif]
