# Supplementary material for: Interleukin 6 is increased in preclinical HNSCC models of acquired cetuximab resistance, but is not required for maintenance of resistance
Source: PLoS One. 2020 Jan 8;15(1):e0227261. doi: 10.1371/journal.pone.0227261 (PMC6948745; doi:10.1371/journal.pone.0227261)
Supplement: S1 Table — (DOCX) [file pone.0227261.s009.docx]

**S1 Table. qPCR primers.**

| **Primer Name** | **Sequence** |
| --- | --- |
| *IL6* forward | GGTACATCCTCGACGGCATCT |
| *IL6* reverse | GTGCCTCTTTGCTGCTTTCAC |
| *IL6R* forward | AGTGTCGGGAGCAAGTTCAG |
| *IL6R* reverse | GGCTGCAAGATTCCACAACC |
| *IL6ST* forward | AGGACCAAAGATGCCTCAAC |
| *IL6ST* reverse | GAATGAAGATCGGGTGGATG |
| *TBP* forward | CCCATGACTCCCATGACC |
| *TBP* reverse | TTTACAACCAAGATTCACTGTGG |
