# Supplementary material for: Interleukin 6 is increased in preclinical HNSCC models of acquired cetuximab resistance, but is not required for maintenance of resistance
Source: PLoS One. 2020 Jan 8;15(1):e0227261. doi: 10.1371/journal.pone.0227261 (PMC6948745; doi:10.1371/journal.pone.0227261)
Supplement: S1 Raw Images — (PDF) [file pone.0227261.s014.pdf]

Fig 2A

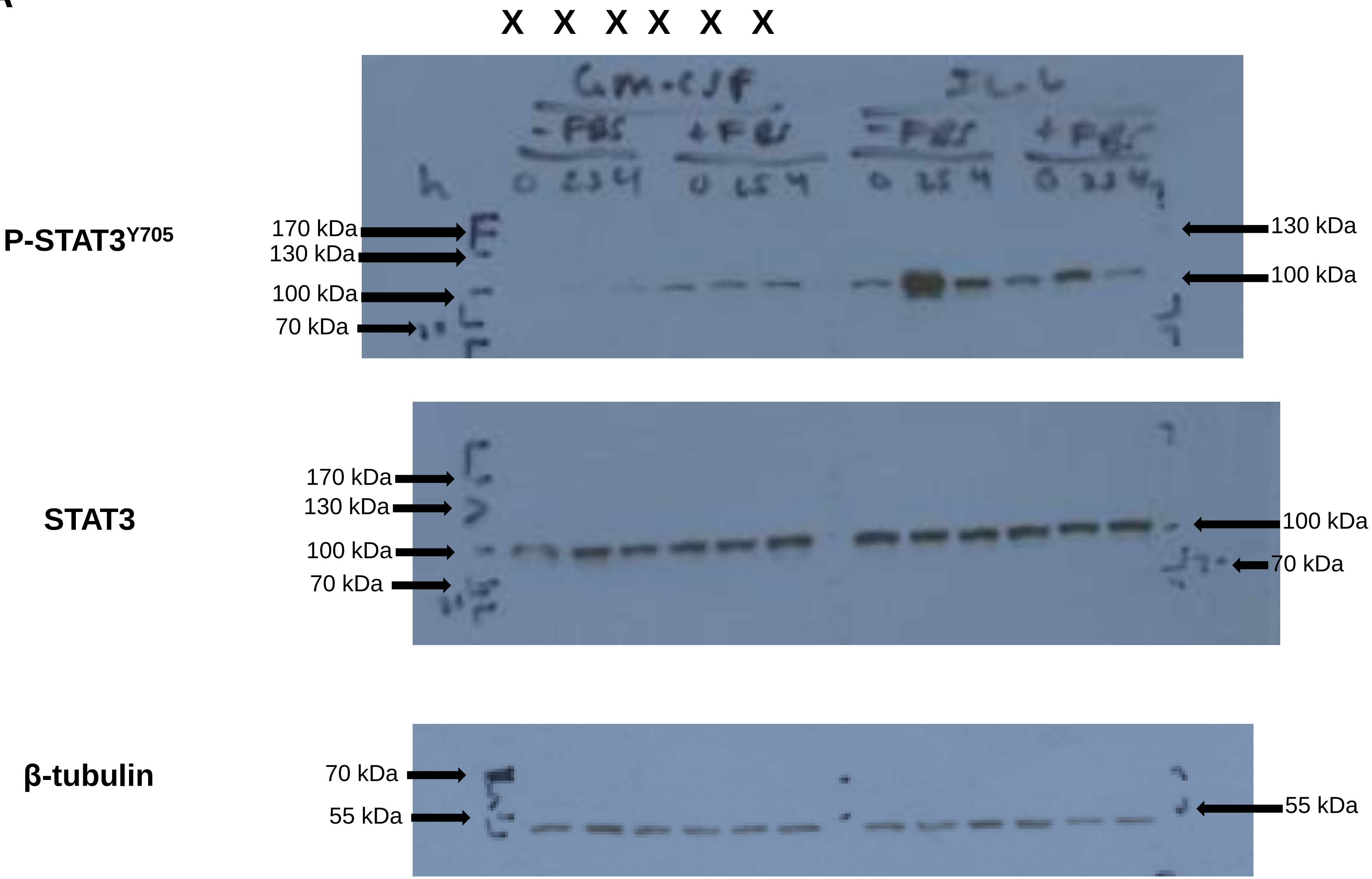

Samples in lanes marked 'X' were for a different project

Fig 4C

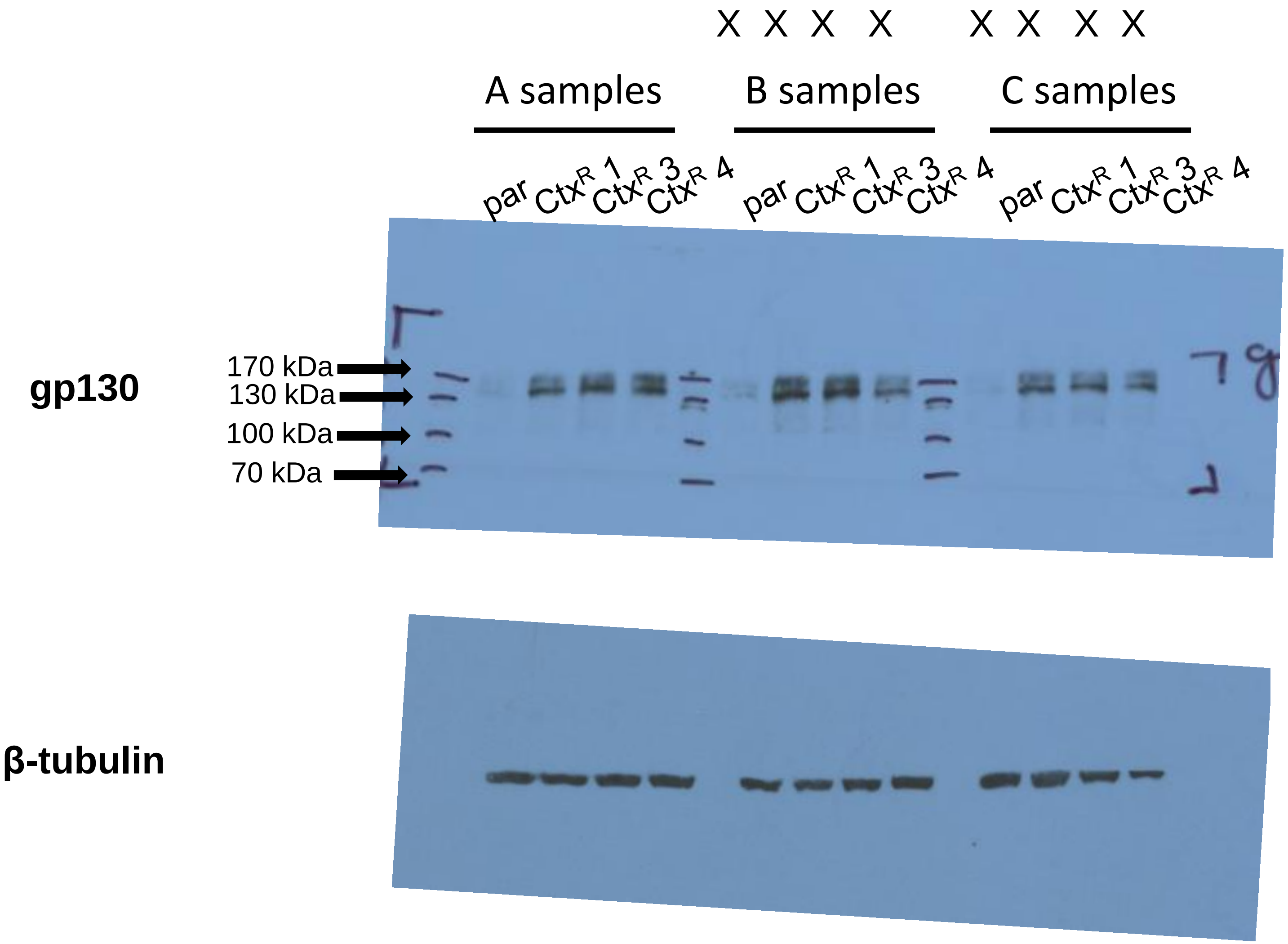

Lanes marked 'X' are biological replicates and were used for quantification (Fig 4D), but not in image in Fig 4C

Fig 5A

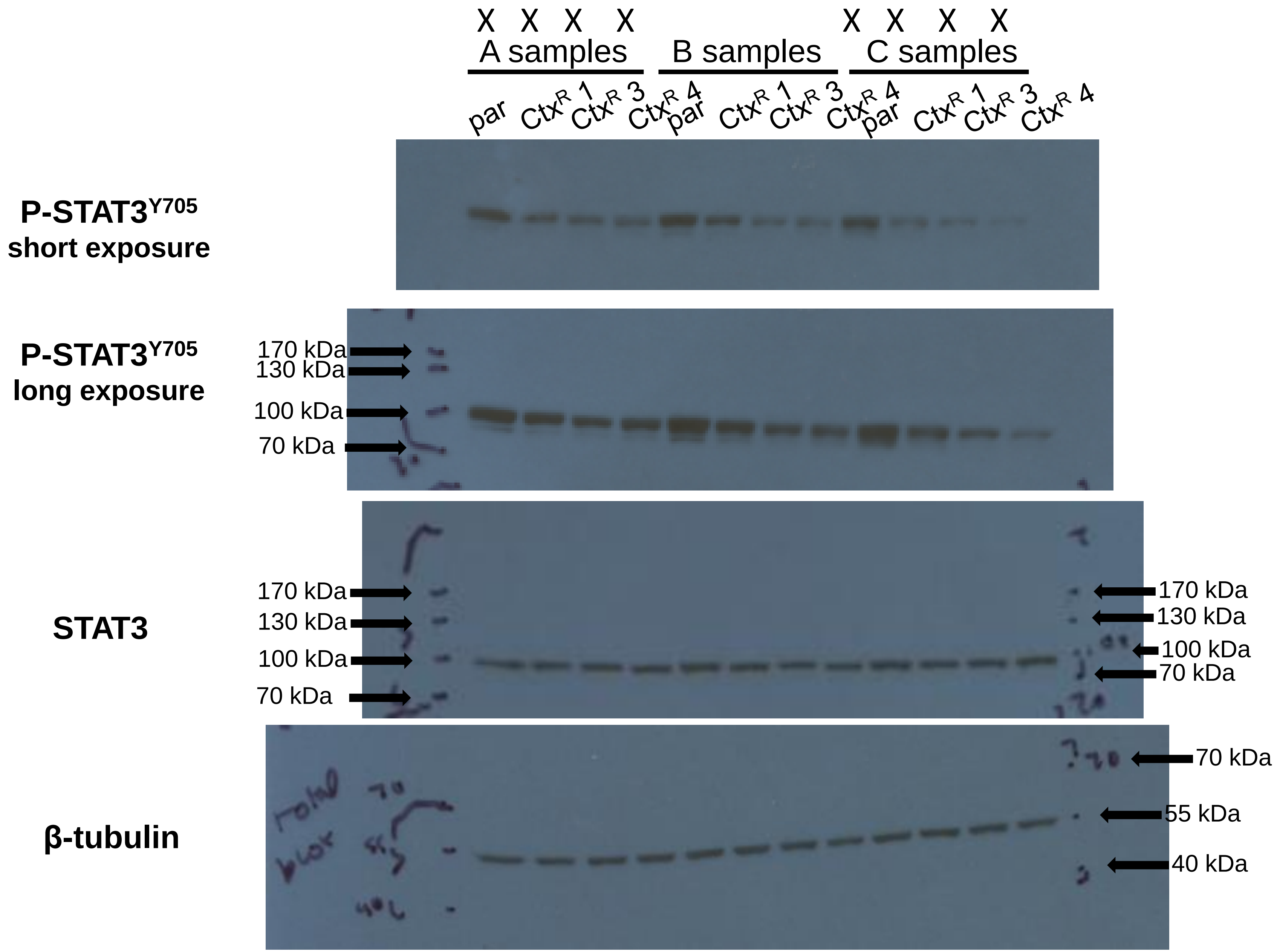

Lanes marked 'X' are biological replicates and were used for quantification (Fig 5B), but not in image in Fig 5A

Fig 5C

P-STAT3^{Y705}
short exposure

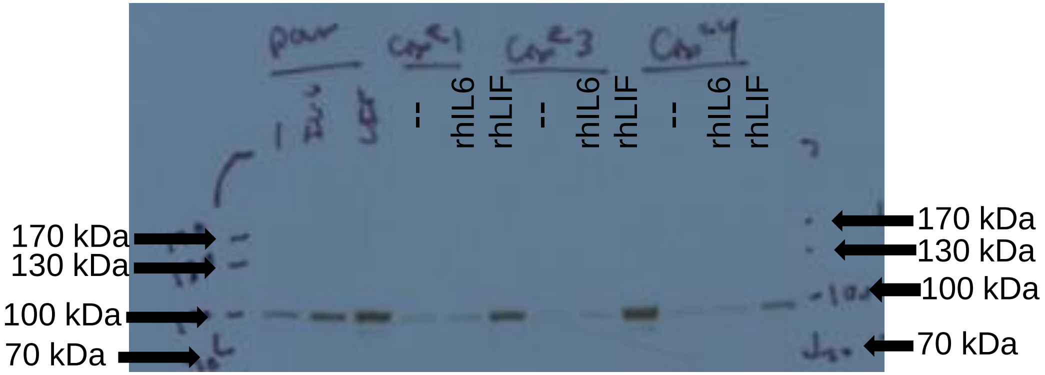

STAT3

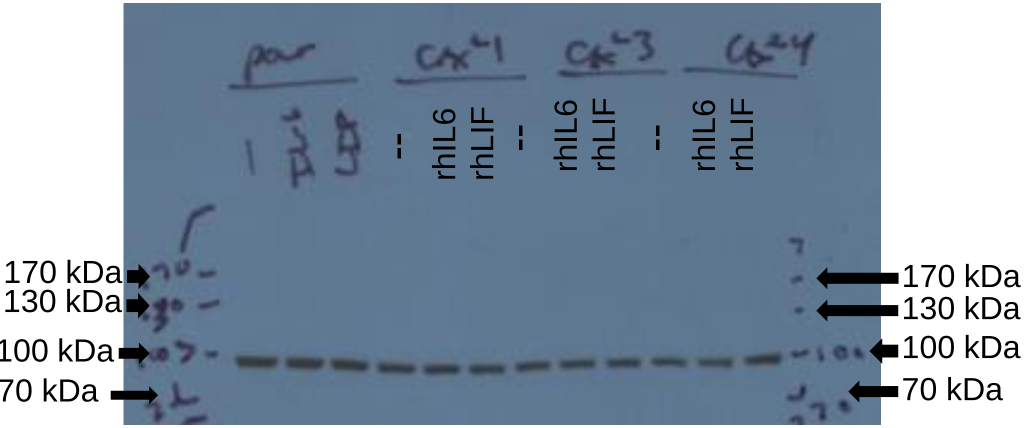

P-STAT3^{Y705} (long exposure) and β-tubulin
Blot was cut at the 70 kDa marker
P-STAT3^{Y705} is on the top
β-tubulin is on the bottom

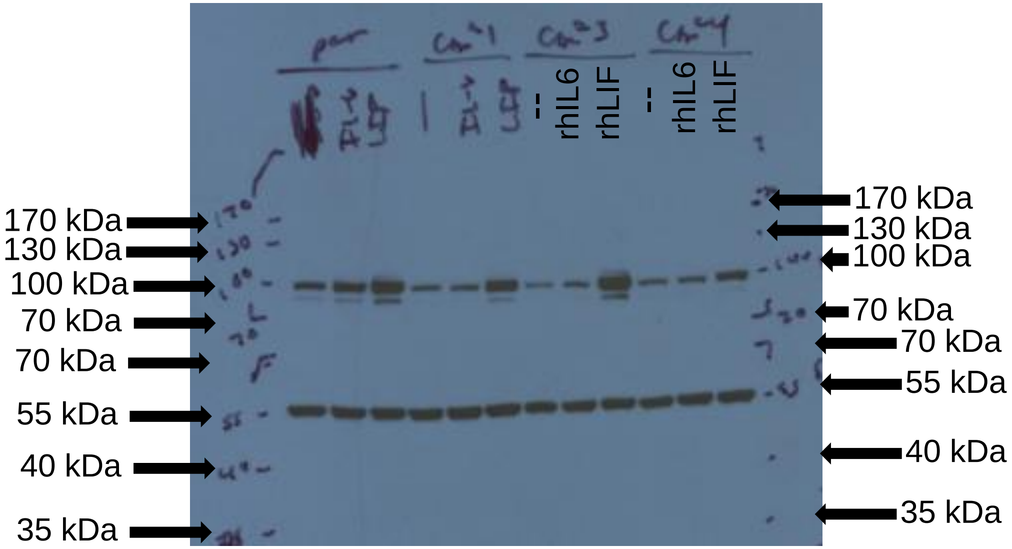

S6 Fig

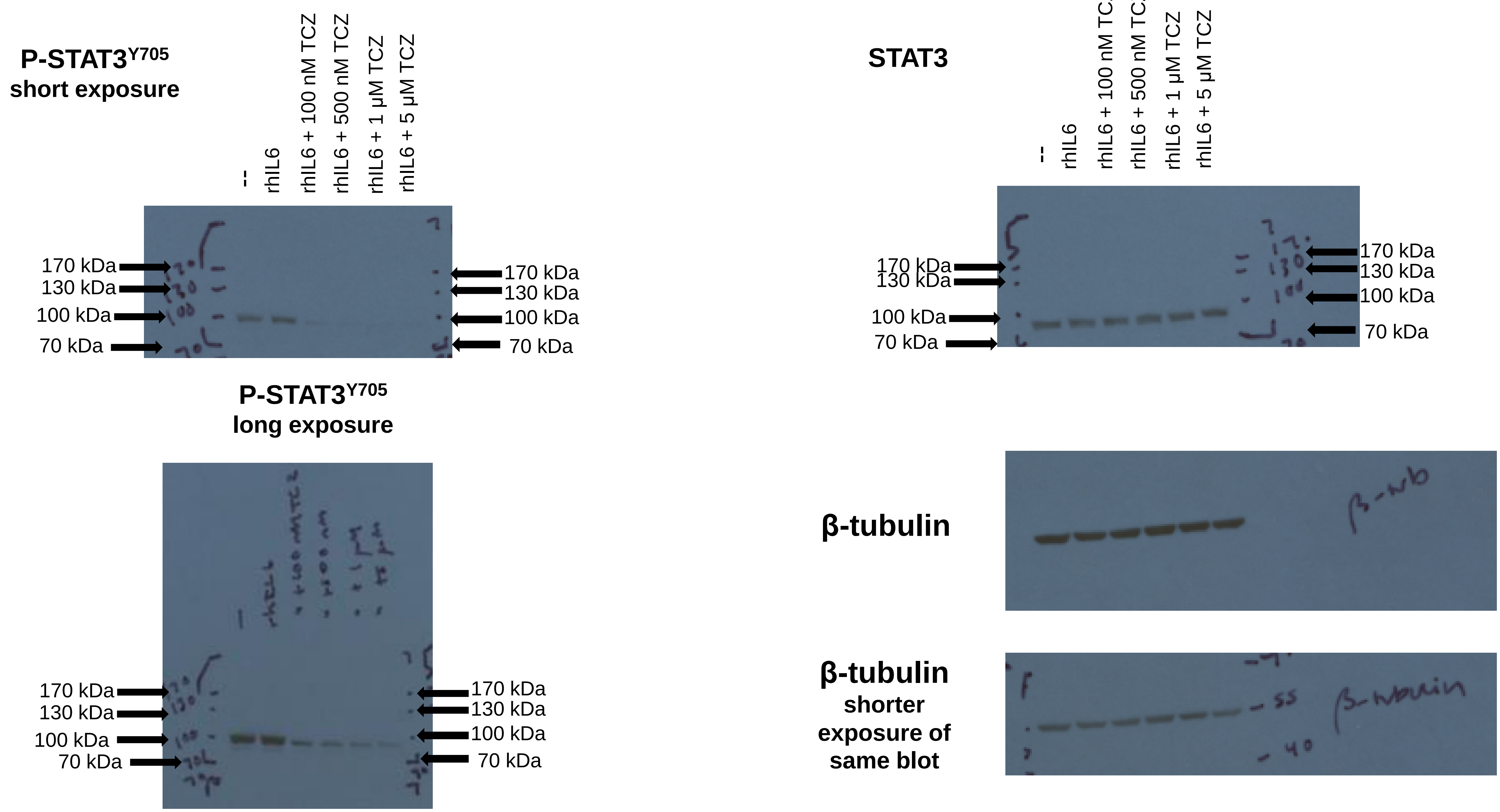
P-STAT3Y705
short exposure
--
rhIL6
rhIL6 + 100 nM TCZ
rhIL6 + 500 nM TCZ
rhIL6 + 1 µM TCZ
rhIL6 + 5 µM TCZ
170 kDa
130 kDa
100 kDa
70 kDa
170 kDa
130 kDa
100 kDa
70 kDa
P-STAT3Y705
long exposure
170 kDa
130 kDa
100 kDa
70 kDa
170 kDa
130 kDa
100 kDa
70 kDa
STAT3
--
rhIL6
rhIL6 + 100 nM TCZ
rhIL6 + 500 nM TCZ
rhIL6 + 1 µM TCZ
rhIL6 + 5 µM TCZ
170 kDa
130 kDa
100 kDa
70 kDa
170 kDa
130 kDa
100 kDa
70 kDa
β-tubulin
β-tubulin
shorter
exposure of
same blot

S7 Fig

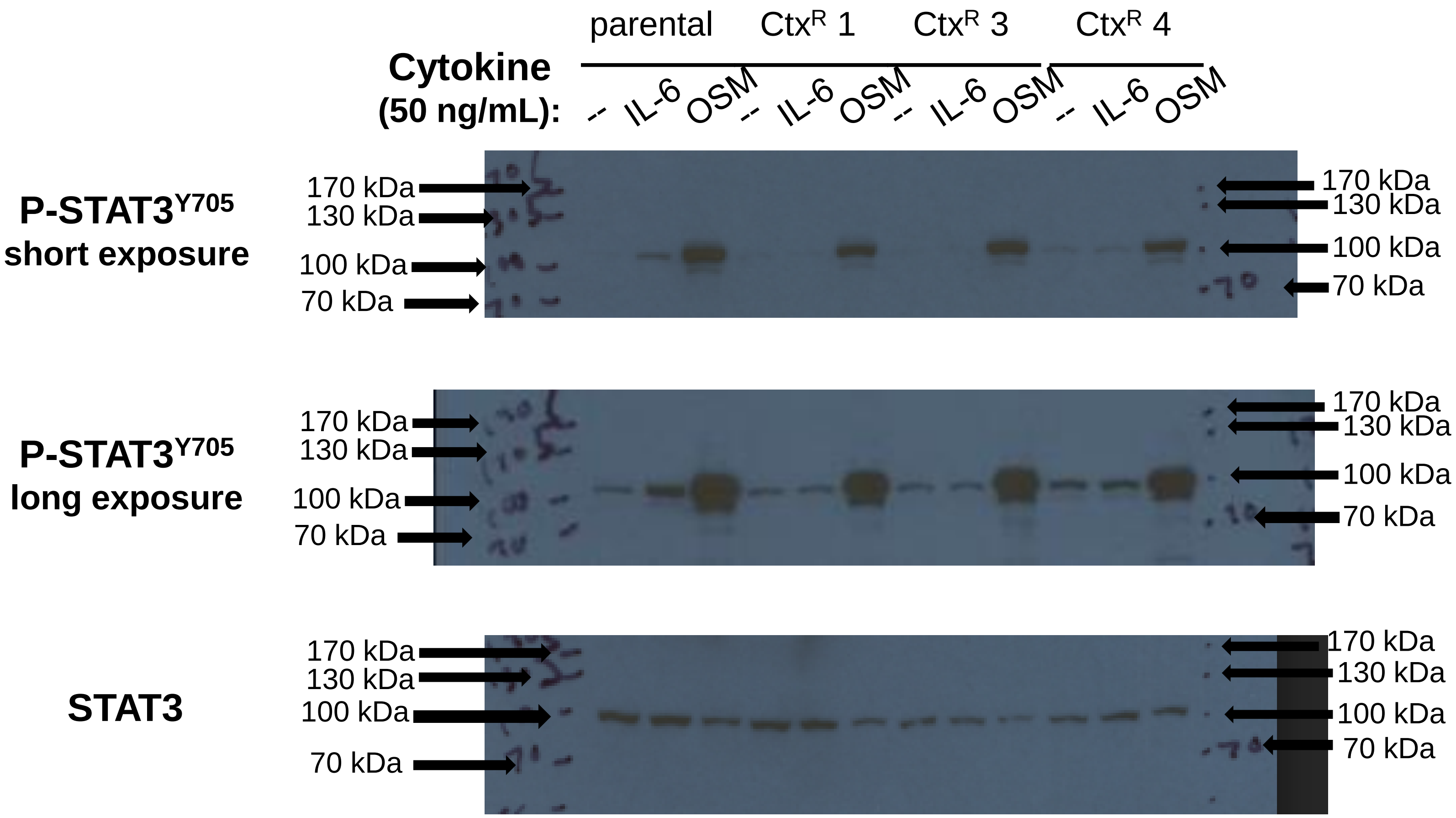
parental
CtxR 1
CtxR 3
CtxR 4
Cytokine
(50 ng/mL):
--
IL-6
OSM
P-STAT3Y705
short exposure
P-STAT3Y705
long exposure
STAT3
170 kDa
130 kDa
100 kDa
70 kDa
